# Impact of Poly(Ester Amide) Structure on Properties and Drug Delivery for Prostate Cancer Therapy

**DOI:** 10.34133/bmef.0025

**Published:** 2023-08-10

**Authors:** Junfu Zhang, Liying Wang, Mengting Ding, Xinru You, Jun Wu, Jun Pang

**Affiliations:** ^1^ Department of Urology, Kidney and Urology Center, Pelvic Floor Disorders Center, The Seventh Affiliated Hospital, Sun Yat-sen University, Shenzhen 518107, China.; ^2^ Department of Hematology, The Seventh Affiliated Hospital, Sun Yat-sen University, Shenzhen 518107, China.; ^3^ Center for Nanomedicine and Department of Anesthesiology, Brigham and Women's Hospital, Harvard Medical School, Boston, MA 02115, USA.; ^4^ Bioscience and Biomedical Engineering Thrust, The Hong Kong University of Science and Technology (Guangzhou), Nansha, Guangzhou 511400, China.; ^5^ Division of Life Science, The Hong Kong University of Science and Technology, Hong Kong SAR, China.

## Abstract

*Objective:* We aim to develop a polymer library consisting of phenylalanine-based poly(ester amide)s (Phe-PEAs) for cancer therapy and investigate the structure–property relationship of these polymers to understand their impact on the drug delivery efficiency of corresponding nanoparticles (NPs). *Impact Statement:* Our study provides insights into the structure–property relationship of polymers in NP-based drug delivery applications and offers a potential polymer library and NP platform for enhancing cancer therapy. *Introduction:* Polymer NP-based drug delivery systems have demonstrated substantial potential in cancer therapy by improving drug efficacy and minimizing systemic toxicity. However, successful design and optimization of these systems require a comprehensive understanding of the relationship between polymer structure and physicochemical properties, which directly influence the drug delivery efficiency of the corresponding NPs. *Methods:* A series of Phe-PEAs with tunable structures was synthesized by varying the length of the methylene group in the diol part of the polymers. Subsequently, Phe-PEAs were formulated into NPs for doxorubicin (DOX) delivery in prostate cancer therapy. *Results:* Small adjustments in polymer structure induced the changes in the hydrophobicity and thermal properties of the PEAs, consequently NP size, drug loading capacity, cellular uptake efficacy, and cytotoxicity. Additionally, DOX-loaded Phe-PEA NPs demonstrated enhanced tumor suppression and reduced side effects in prostate tumor-bearing mice. *Conclusion:* Phe-PEAs, with their finely tunable structures, show great promise as effective and customizable nanocarriers for cancer therapy.

## Introduction

Prostate cancer, the second most common male malignancy globally, has seriously jeopardized the life and health of patients [1–3]. Currently, chemotherapy remains an important option for patients with advanced prostate cancer, especially those with castration-resistant prostate cancer [4–7]. However, traditional chemotherapy faces many challenges, such as unavoidable drug resistance, limited target efficiency, low specificity, rapid clearance, and serious side effects—greatly contributing to the unsatisfactory therapeutic efficacy [6–10]. Therefore, it is critical to develop more effective and safer therapy for prostate cancer.

Drug delivery systems have emerged as a promising alternative to traditional cancer therapy due to their unique advantages in enhancing specificity, prolonging circulation time, improving therapeutic outcomes, and reducing side effects [11–16]. Polymeric nanoparticles (PNPs) are particularly well-studied as delivery systems for diagnostics and drug delivery [17–19], as they pose good biocompatibility, biodegradability, and nonimmunogenicity, as well as adjustable physicochemical properties [20–23]. Additionally, PNPs offer great potential in encapsulating various therapeutic agents, releasing payloads in a sustained or controlled manner at targeted sites, and enhancing therapeutic performance [24–28]. However, the design of these systems requires a thorough understanding of the relationship between polymer structures and physicochemical properties, which can affect the drug delivery efficiency of the corresponding NPs. There have been relatively few studies on this aspect.

Among the various polymer drug delivery systems, poly(ester amide)s (PEAs) are a promising synthetic polymer family that might be a desirable candidate for exploring the structure–property relationship of polymers. PEAs are mainly synthesized from 3 basic building blocks: amino acids, diacids, and diols [29,30]. By adjusting the types and ratios of these monomers, the physicochemical properties of PEAs can be finely regulated. Specifically, the choice of amino acid monomers can affect the overall hydrophilicity/hydrophobicity and electrical properties of the resulting PEAs, while the types of diacid and diol monomers can alter the molecular weight and thermodynamic properties, and collectively influence the interactions between the polymers and drugs, as well as the interactions between the materials and cells, ultimately affecting the overall performance of PEAs as drug delivery systems [31–34]. Therefore, the ability to tailor the physicochemical properties of PEAs by modifying the monomer structure enables the development of highly customized polymers that can meet specific therapeutic needs, such as drug delivery systems for prostate cancer therapy.

**Fig. 1.**
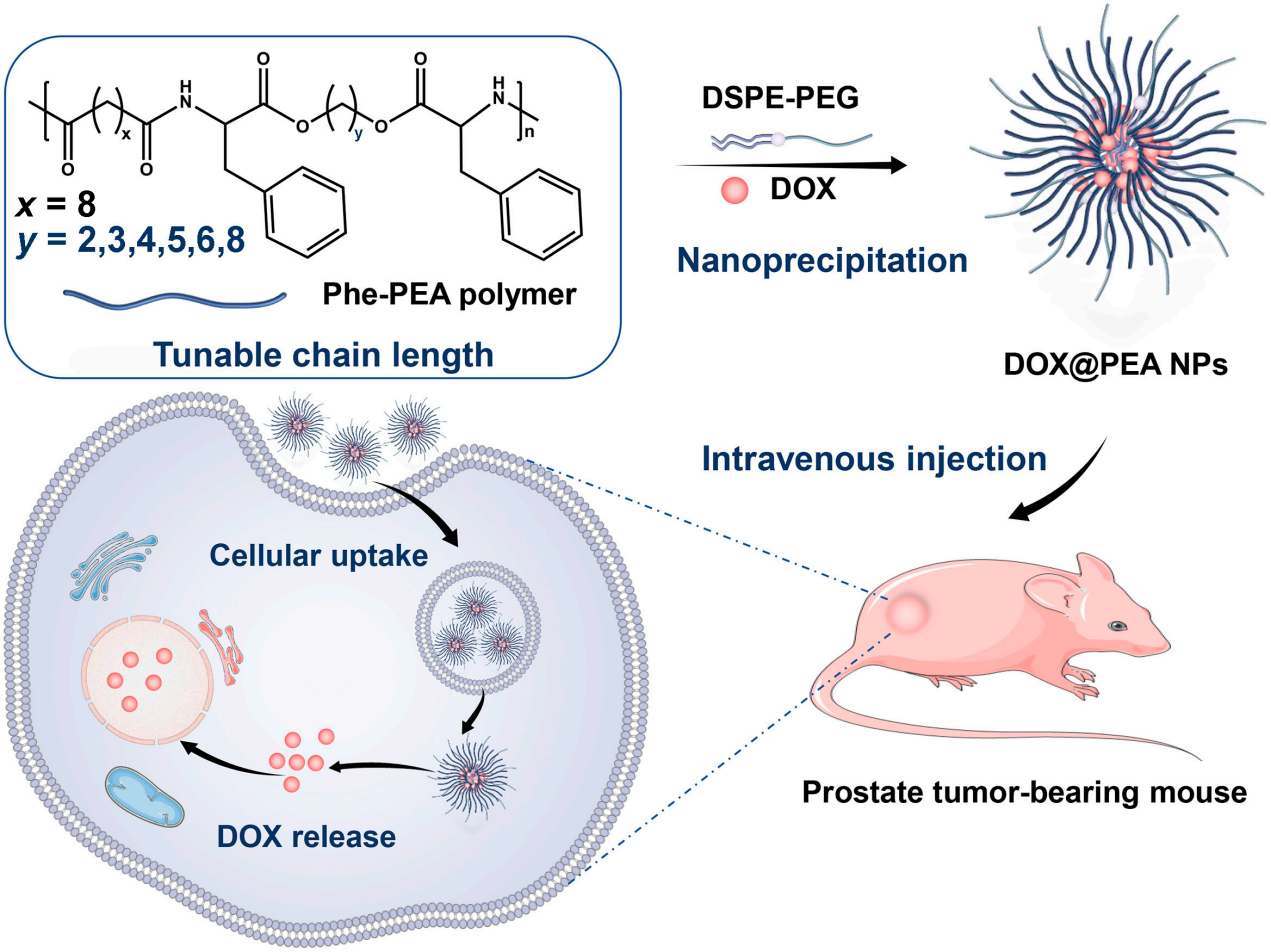
Construction of Phe-PEA polymer-based nanoplatform for drug delivery.

In this study, with the aim of investigating the structure–property relationship and developing efficient delivery systems for prostate therapy, we selected nontoxic L-phenylalanine (Phe) as one of the building blocks to construct a library of Phe-based PEA (Phe-PEA) polymers via solution polycondensation. By slightly adjusting the length of the methylene group in the diol part of the polymers, we obtained a series of Phe-PEAs and systemically characterized their physicochemical properties. We found that the tiny adjustment in polymer structure could induce changes in molecular weight, hydrophobicity, and thermal property of polymers. Subsequently, Phe-PEAs were used to construct a nanoparticle (NP) platform via the nanoprecipitation method and encapsulated doxorubicin (DOX) for prostate cancer therapy (Fig. 1). We characterized the properties and in vitro performance of the corresponding NPs, including NP size, stability, drug loading capacity, cellular uptake efficacy, and cytotoxicity. In particular, 2 types of DOX-loaded PEA NPs (DOX@PEA NPs) with the highest drug loading capacity and preferential cytotoxicity were chosen to conduct an in vivo anticancer study. As expected, the DOX@PEA NPs all showed remarkable antitumor performance and low systemic toxicity against prostate cancer, indicating the great promise of Phe-PEA polymers as drug delivery systems.

## Results and Discussion

### Synthesis and characterization of Phe-PEA polymers

**Fig. 2.**
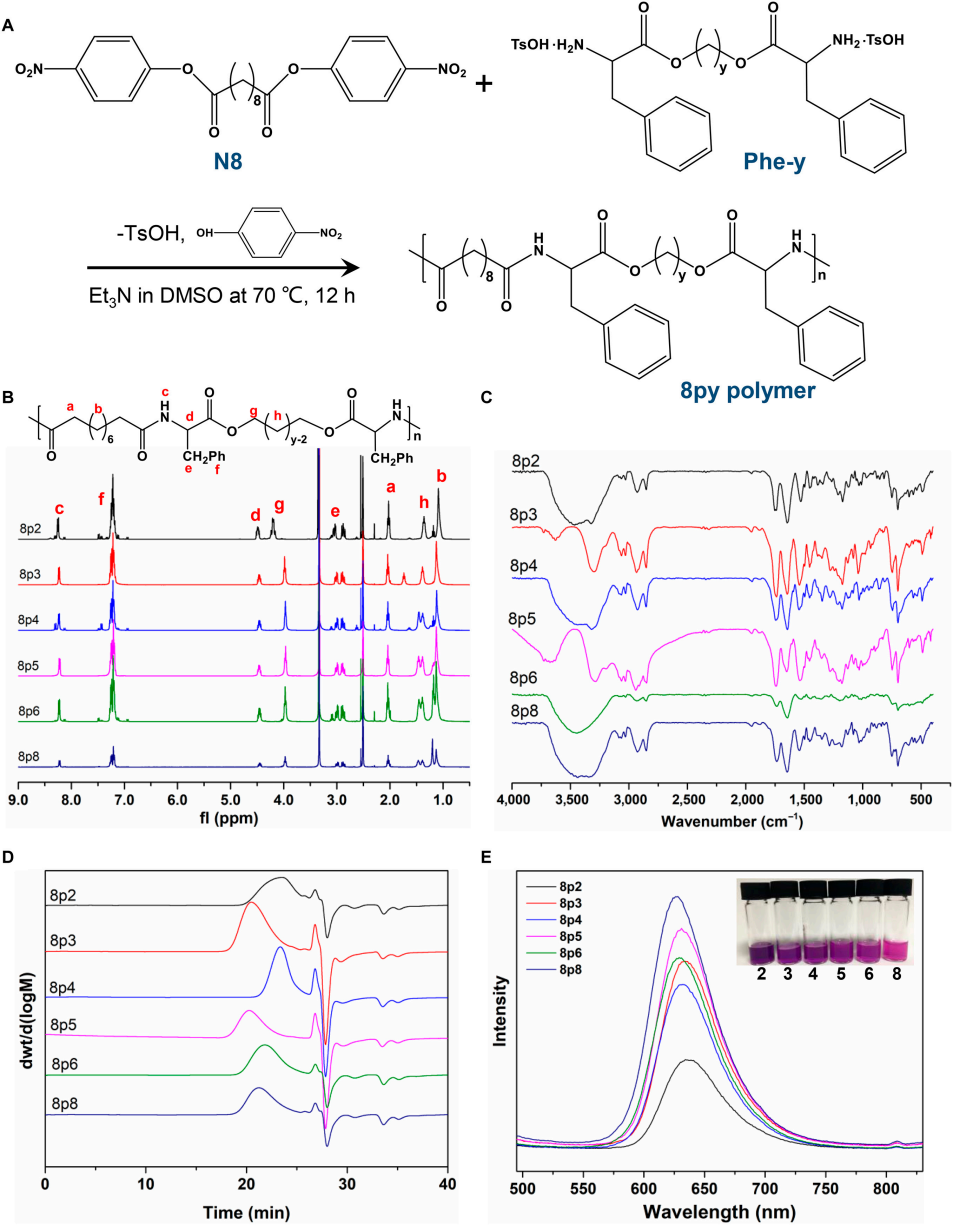
Synthesis and characterization of the 8py polymers. (A) Synthetic route of 8py polymers. (B) ^1^H-NMR spectra of 8py polymers. (C) FT-IR spectra of 8py polymers. (D) GPC spectra of 8py polymers. (E) Hydrophobicity test of 8py polymers.

PEA with flexible combinations and tunable properties is an attractive material for drug carrier development. PEA polymers are mainly composed of 3 major building blocks: amino acid, dicarboxylic acid, and fatty diol. In this study, we chose Phe as the amino acid moiety and adjusted the type of diols to synthesize a series of monomers, denoted as Phe-y, where y means the methylene group in diols. Diacid moiety was determined as di-*p*-nitrophenyl sebacate (N8). The chemical structures of Phe-y and N8 were confirmed by ^1^H-nuclear magnetic resonance (^1^H-NMR), as shown in Figs. S1 to S7. Next, a family of Phe-PEAs, designated as 8py polymers, were synthesized through solution polycondensation following the protocols of previous reports (Fig. 2A) [35]. The chemical structures of the synthesized 8py polymers were validated by ^1^H-NMR and Fourier-transform infrared spectroscopy (FT-IR). As evidenced by Fig. 2B, the corresponding proton peaks in 8py polymers were indicated in the spectrum. Additionally, as shown in Fig. 2C, C=O stretch peaks from the original Phe and diacids were observed at 1,720 and 1,670 cm^−1^, respectively. These results collectively confirmed the successful polymerization of 8py polymers.

Subsequently, we determined the molecular weight (MW) of the 8py polymers using gel permeation chromatography (GPC), as depicted in Fig. 2D and Table S1. Interestingly, an odd–even effect was observed in the MW of the polymers, where 8py polymers with an odd “y” value displayed higher MW and polydispersity index (PDI) values, while the MW of 8py polymers with an even “y” value mainly ranged from 20,000 to 40,000. Additionally, we investigated the thermal properties of the 8py polymers using differential scanning calorimetry, as shown in Table S1. The glass transition temperature (Tg) of the polymers ranged from 19.1 to 42.2 °C and also exhibited an odd–even effect. Correspondingly, polymers with an odd “y” value had a higher Tg, which may be attributed to differences in crystallinity and MW between the 2 types of polymers. Next, the hydrophobicity of the polymers was investigated by encapsulating Nile red, a dye that exhibits environment-dependent fluorescence. Under the conditions of equivalent dye feeding, polymers with higher hydrophobicity allow more retention of Nile red, thereby leading to enhanced fluorescence intensity [36]. As shown in Fig. 2E, the 8py polymer with a longer methylene chain generally resulted in stronger hydrophobicity.

### Fabrication and characterization of Phe-PEA NPs

**Fig. 3.**
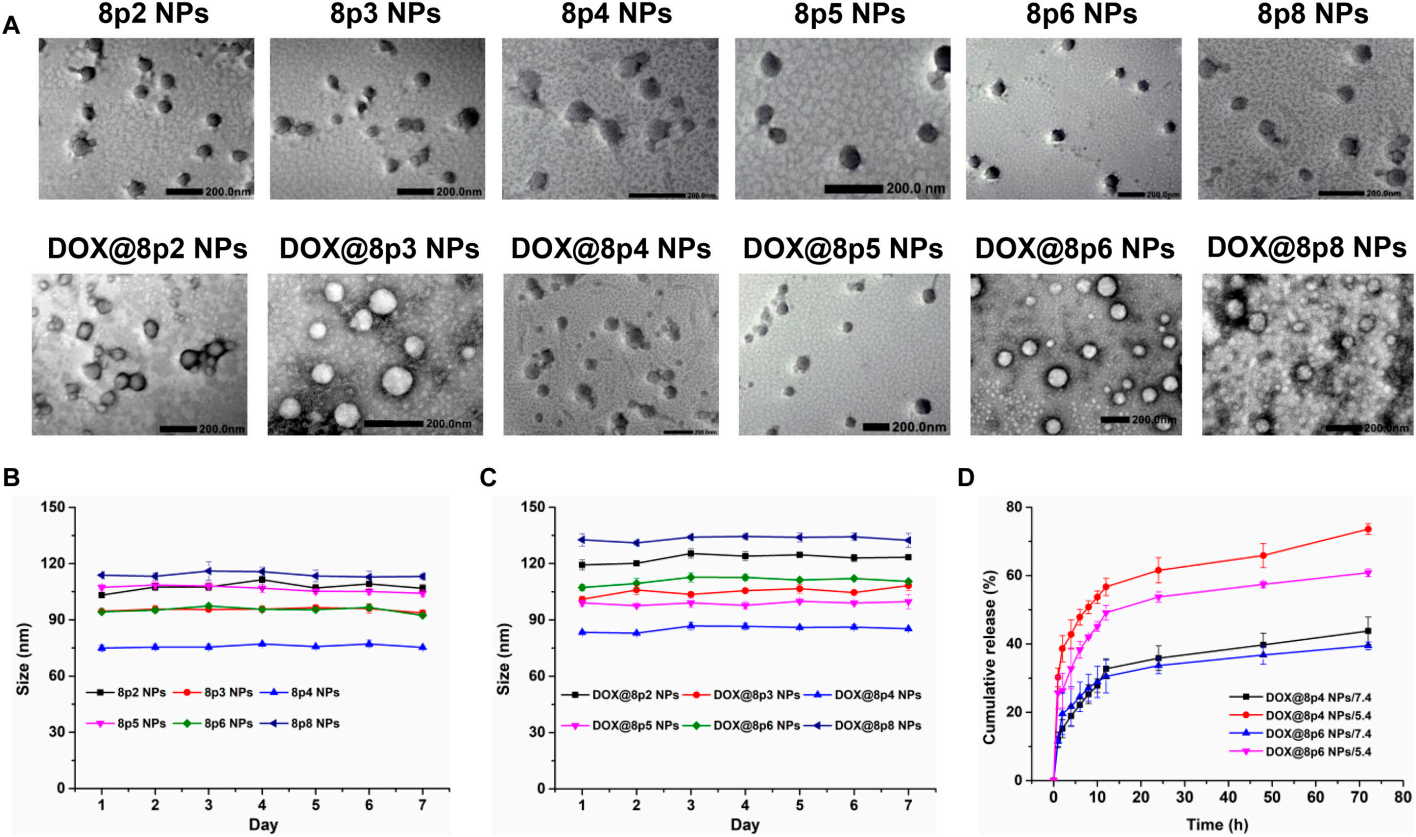
Characterization of 8py NPs and DOX@8py NPs. (A) Representative TEM images of 8py NPs and DOX@8py NPs. Scale bar: 200 nm. Stability test of 8py NPs (B) and DOX@8py NPs (C) in PBS for 7 days. (D) Drug release profiles of DOX@8py NPs in PBS with pH values of 5.4 and 7.4.

Next, the 8py polymers were utilized to develop a nanoplatform for the delivery of DOX. The commonly used nanoprecipitation method was employed to fabricate both blank 8py NPs and DOX@8py NPs. In the presence of amphiphilic DSPE-PEG, the 8py polymers self-assembled into NPs, creating a hydrophobic interior that facilitated the encapsulation of DOX. Blank 8py NPs displayed a narrow size distribution ranging from 75 to 113 nm, while DOX incorporation slightly increased the NP diameter from 83 to 133 nm, as confirmed by dynamic light scattering (DLS) results (Table 1 and Fig. S8). Additionally, the morphology of the NPs was visualized by a transmission electron microscope (TEM). All the NPs exhibited well-defined spherical structures with sizes coinciding with the DLS results (Fig. 3A).

**Table.** Characterization results of blank 8py NPs and corresponding DOX@8py NPs.

NPs	Blank 8py NPs		DOX@8py NPs			
Polymer	Size (nm)	PDI	Size (nm)	PDI	DLC (%)	DLE (%)
8p2	106.8 ± 1.9	0.07 ± 0.03	119.2 ± 2.7	0.13 ± 0.02	13.57 ± 0.07	67.85 ± 0.37
8p3	93.6 ± 1.5	0.12 ± 0.07	100.9 ± 1.7	0.07 ± 0.03	13.72 ± 0.08	68.61 ± 0.42
8p4	75.2 ± 2.7	0.12 ± 0.02	83.3 ± 0.7	0.10 ± 0.01	14.35 ± 0.15	71.77 ± 0.77
8p5	104.1 ± 1.7	0.11 ± 0.05	99.0 ± 1.8	0.12 ± 0.03	13.76 ± 0.13	68.82 ± 0.66
8p6	92.3 ± 0.6	0.13 ± 0.03	107.2 ± 1.7	0.10 ± 0.04	14.30 ± 0.09	71.53 ± 0.45
8p8	113.1 ± 1.3	0.14 ± 0.02	132.7 ± 3.3	0.19 ± 0.03	13.90 ± 0.07	69.50 ± 0.36

DLC, drug loading capacity; DLE, drug loading efficiency.

Subsequently, the stability of 8py NPs and DOX@8py NPs was assessed by monitoring particle size using DLS over 7 days in PBS and PBS containing serum. As shown in Fig. 3B and C and Fig.S9, most blank 8py NPs and DOX@8py NPs all exhibited good stability without substantial fluctuation in size over a long period. However, it is noteworthy that the particle size of DOX@8p2 NPs exhibited a substantial increase in a serum environment, rendering them unsuitable for in vivo applications. The DOX-loading capacity of DOX@8py NPs was determined by a fluorescence spectrophotometer, with results presenting in Table. Obviously, no significant difference was observed in DLC among all the DOX-loaded NPs. DOX@8p4 and DOX@8p6 NPs exhibited slightly improved DLC values of 14.35% ± 0.15% and 14.30% ± 0.09%, respectively. Overall, with suitable particle size, long-term stability, and acceptable drug loading capacity, Phe-PEA NPs can serve as a promising NP platform for drug delivery.

### Drug release behavior of DOX@8py NPs

DOX@8p4 NPs and DOX@8p6 NPs, which had the highest drug loading capacity, were selected for investigating in vitro DOX release behavior. The release study was conducted in 2 separate buffers: PBS at pH 7.4, mimicking physiological conditions, and PBS at pH 5.4, representing the acidic tumor microenvironment. As shown in Fig. 3D, at pH 7.4, DOX@8py NPs exhibited relatively slow DOX release with a cumulative release of ~40% at 72 h. However, under acidic conditions, DOX was more rapidly released from the NPs and reached a higher cumulative release, 73.62% for DOX@8p4 NPs vs. 60.84% for DOX@8p6 NPs. This may be attributed to the fact that the ester bonds in the 8py polymers could be broken down more easily under acidic conditions, thereby inducing the rapid disintegration of the NPs. It is noticeable that DOX@8p4 NPs exhibited a more rapid DOX release speed and a higher cumulative DOX release than DOX@8p6 NPs under the 2 conditions, which might be caused by the difference in the biodegradable speed of the polymers. Overall, DOX@8py NPs could achieve more effective drug release in a simulated tumor environment, further indicating the great potential of 8py NPs as a drug delivery platform.

### In vitro cytotoxicity of DOX@8py NPs

**Fig. 4.**
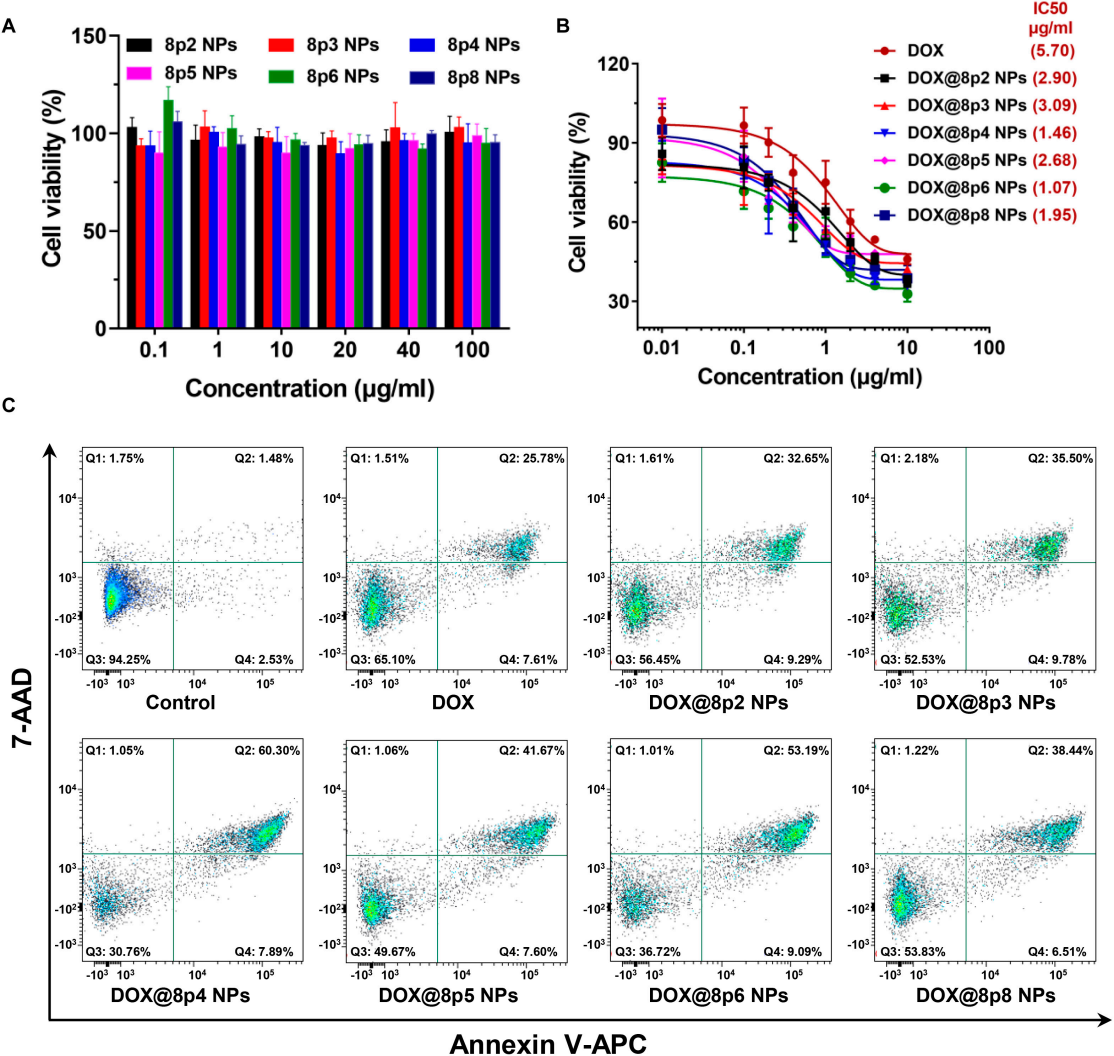
In vitro anticancer evaluation of DOX@8py NPs. (A) Cell viability of LNCaP cells after incubation with 8py NPs for 24 h. (B) Cell viability of LNCaP cells after incubation with DOX@8py NPs across different concentrations for 24 h. (C) Flow cytometry analysis for the apoptosis of LNCaP cells induced by DOX and DOX@8py NPs.

The cytotoxicity of blank 8py NPs and DOX@8py NPs against LNCaP cells was assessed by 3-(4,5-dimethylthiazol-2-yl)-2,5-diphenyltetrazolium bromide (MTT) assay in vitro*.* As displayed in Fig. 4A, all the blank 8py NPs showed little toxicity to LNCaP cells and the cells maintained a very high cell viability even at a very high NP dosage, indicating the good biocompatibility of 8py NPs as drug delivery carriers. In contrast, DOX and DOX@8py NPs were able to elicit pronounced and concentration-dependent suppression of the cells (Fig. 4B). More importantly, all DOX@8py NPs exhibited a higher half-maximal inhibitory concentration (IC_50_) value than free DOX, suggesting the superior inhibition effect of DOX@8py NPs on cell proliferation. Specifically, DOX@8p4 NPs and DOX@8p6 NPs with minimal IC_50_ values of 1.46 and 1.07 μg/ml might be the preferable nanoformulations among DOX@8py NPs.

### Cell apoptosis analysis

To further evaluate the cell apoptosis effect of DOX@8py NPs on LNCaP cells, we next performed an apoptosis assay. After treatment with free DOX and DOX@8py NPs, LNCaP cells were collected for flow cytometry analysis. As shown in Fig. 4C and Fig. S10, only a very small proportion of apoptotic cells were observed in the control group. Compared to free DOX with a total apoptosis rate of 33.4% ± 1.9%, DOX@8py NPs all increased cell apoptosis, among which DOX@8p4 NPs and DOX@8p6 NPs exerted more noticeable apoptosis effects, leading to total apoptosis rates of 66.2 ± 4.6% and 64.0 ± 3.4%, respectively. Therefore, 8py NPs as nanocarriers could effectively enhance the in vitro anticancer activity of DOX against prostate cancer.

### Cellular uptake and internalization of DOX@8py NPs

**Fig. 5.**
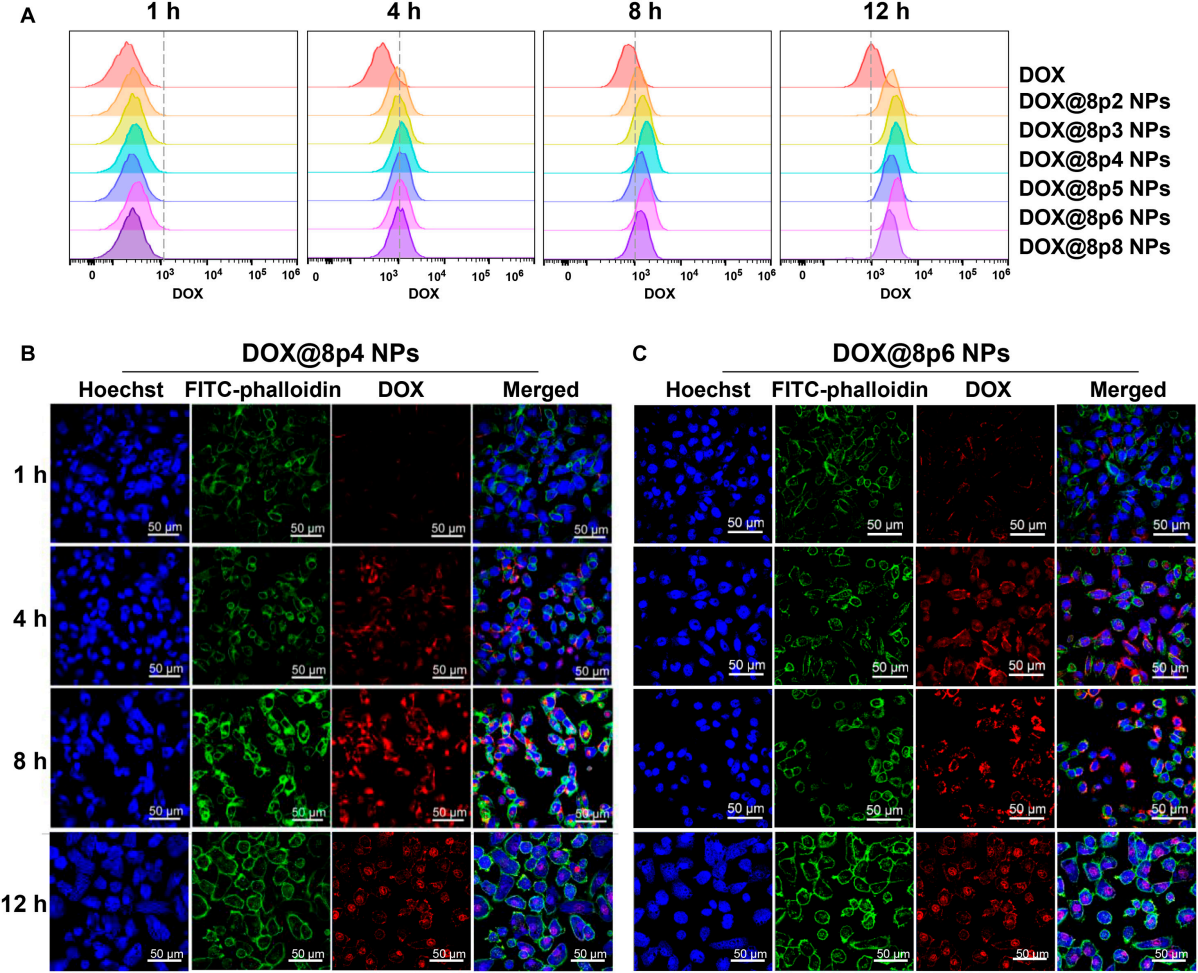
Cellular uptake and internalization of DOX@8py NPs on LNCaP cells. (A) Flow cytometry analysis of DOX@8py NPs uptake after treatment for 1, 4, 8, and 12 h. (B and C) Representative CLSM images of LNCaP cells after incubation with DOX@8p4 NPs (B) and DOX@8p6 NPs (C) for varying periods (1, 4, 8, and 12 h). Blue represents nuclei labeled with Hoechst 33342, green represents FITC-phalloidin staining of the cytoskeleton, and red represents DOX fluorescence. Scale bar: 50 μm.

Next, the cellular uptake efficiency of DOX@8py NPs by prostate cancer cells were investigated by flow cytometer analysis. The results displayed a time-dependent trend as the DOX intensity from both DOX and DOX@8py NPs within cells increased with time, as depicted in Fig. 5A and Fig. S11. Notably, compared to free DOX, all DOX@8py NPs significantly enhanced drug uptake. Among them, DOX@8p4 NPs and DOX@8p6 NPs exhibited a relatively higher uptake level at 8 h and 12 h, and were therefore selected for further investigation of the cellular internalization of DOX@8py NPs by cancer cells. After incubation with the DOX@8py NPs for different times, the cells were observed using confocal laser scanning microscopy (CLSM). The red fluorescence from the DOX was initially weak, which significantly increased and primary located in the cytoplasm at 4 h, as shown in Fig. 5B and C. As time extended to 8 h, the colocalization between DOX and nuclei was observed and almost all the DOX molecules were transferred into nuclei at 12 h, indicating that DOX@8py NPs could efficiently internalize by cancer cells and reach the target nuclei to exert an anticancer effect. To further accurately determine the DOX levels in cells, LNCaP cells incubated with DOX@8p4 NPs and DOX@8p6 NPs were also detected using high-performance liquid chromatography with a fluorescence detector. As shown in Fig. S12, the cellular DOX levels were low within 1 h, while with increasing time to 4 h, the DOX levels were significantly elevated. However, prolonged incubation time failed to further enhance cellular uptake, which may be attributed to the inhibitory effects of DOX@8py NPs with a higher DOX concentration on cancer cells. Taken together, these results indicate that DOX@8py NPs could achieve effective uptake and internalization by LNCaP cells, indicating the great potential of DOX@8py NPs for prostate cancer treatment.

### In vivo anticancer performance of DOX@8py NPs

**Fig. 6.**
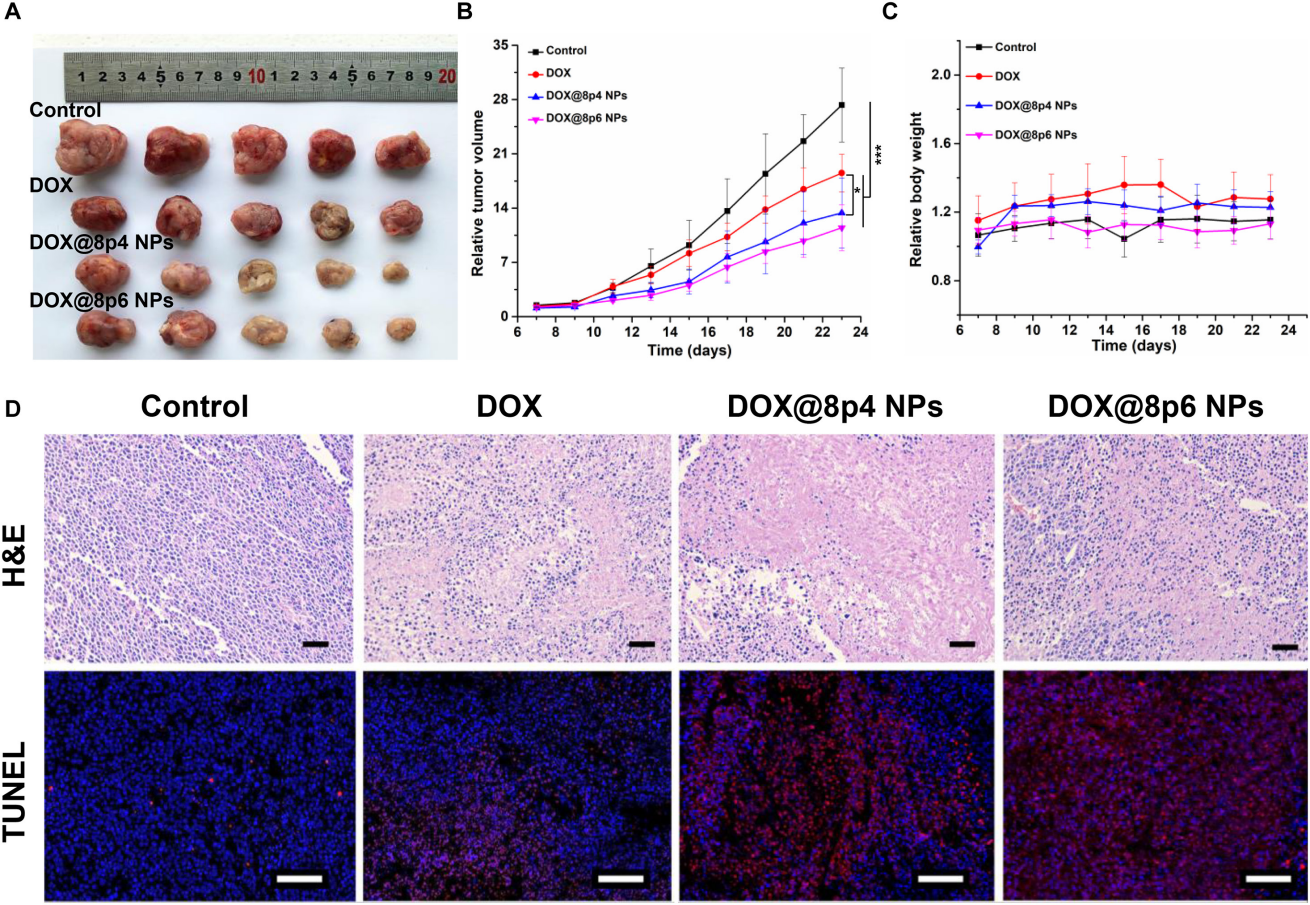
In vivo anti-tumor efficacy of DOX@Phe-PEA NPs in LNCaP tumor-bearing mice. (A) Images of tumor tissues extracted from the mice after treatment with normal saline (control group), DOX (10 mg/kg), DOX@8p4 NPs, and DOX@8p6 NPs (equivalent DOX dose: 10 mg/kg). (B and C) Growth trends in tumor volume (B) and body weight (C) of 4 groups during treatment period. (D) Representative H&E and TUNEL images of tumor tissues in 4 groups. Scale bar: 100 μm. *P < 0.05; ***P < 0.001.

After carefully evaluating the drug loading capacity, in vitro anticancer effect, and cellular uptake efficiency, DOX@8p4 NPs and DOX@8p6 NPs were deemed as the optimal candidates to assess the in vivo anticancer performance of DOX@8py NPs. When the tumors grew to a volume of approximately 100 mm^3^, LNCaP xenograft-bearing mice were treated with various formulations, including saline, DOX, DOX@8p4 NPs, and DOX@8p6 NPs via intravenous administration. The results in Fig. 6A and B demonstrated that the control group showed rapid and aggressive tumor growth, while free DOX successfully inhibited the tumor growth. Moreover, compared to the DOX group, the DOX@8py NPs displayed significantly better suppression of tumor growth, which could be attributed to the superior advantages of NPs in drug delivery. Although DOX@8p6 NPs resulted in the smallest tumor volume, there was no significant difference in tumor suppression between DOX@8p4 NPs and DOX@8p6 NPs. Importantly, the administration of DOX@8py NPs did not result in any weight loss and exhibited a growth trend similar to that of the control group, indicating the biosafety of DOX@8py NPs (Fig. 6C).

To evaluate the therapeutic outcomes of DOX@8py NPs, we also conducted hematoxylin and eosin (H&E) staining and terminal deoxynucleotidyl transferase-mediated deoxyuridine triphosphate-nick end labeling (TUNEL) assay on tumor tissues. As displayed in Fig. 6D, the H&E images showed that tumor tissue in the control group retained a well-organized structure, while the DOX and DOX@8py NP groups exhibited a loosely organized structure and extensive cell death. Additionally, the intensity of red fluorescence in TUNEL images could reveal the extent of apoptosis after different treatments. Obviously, DOX and DOX@8py NPs both induced apoptosis in tumor tissues, where DOX@8py NPs resulted in a more remarkable apoptosis effect. Taken together, these results further validated that DOX@8py NPs could cause pronounced inhibition on prostate cancer cells and elicit anticancer activity superior to that of free DOX.

### In vivo biosafety of the DOX@8py NPs

**Fig. 7.**
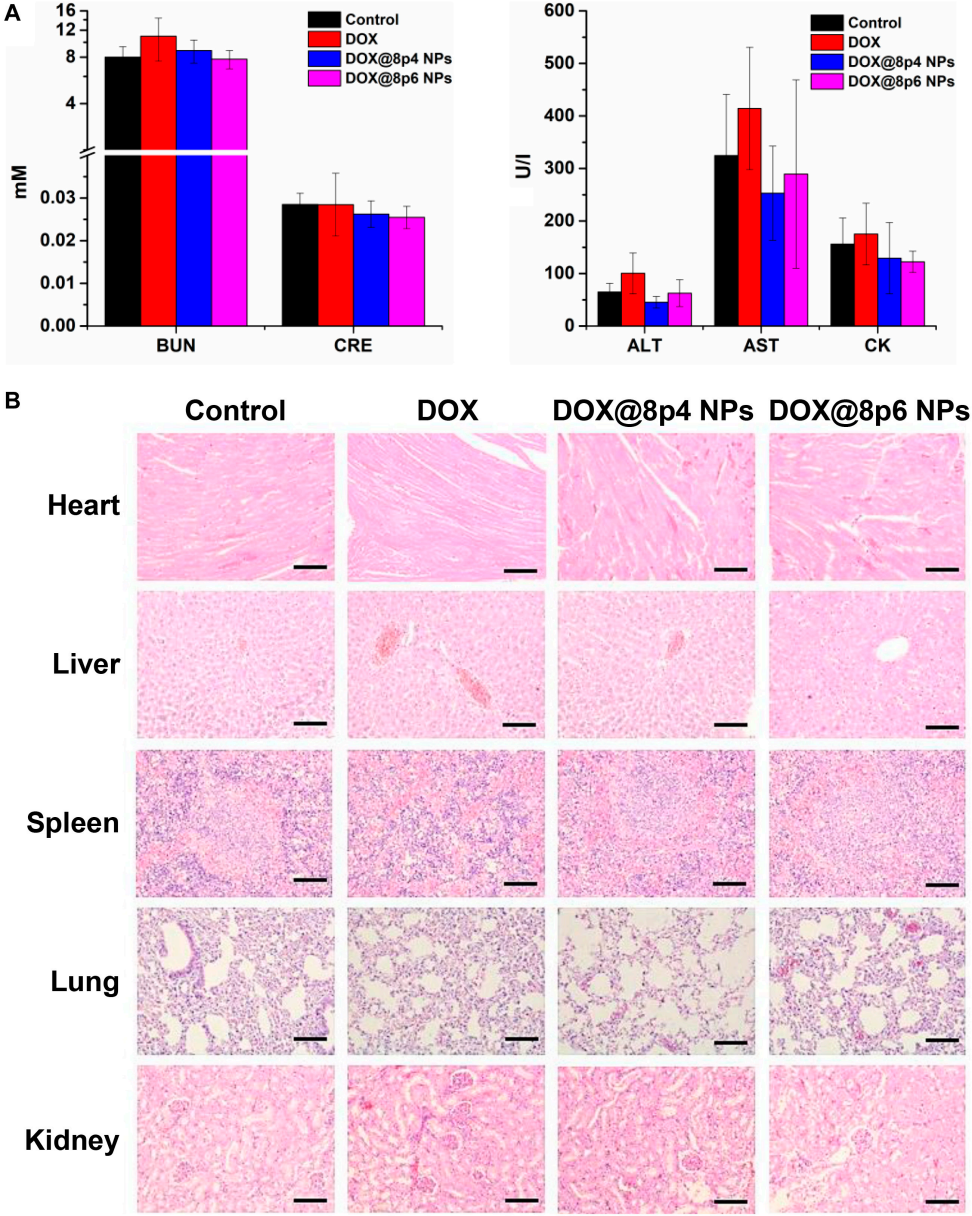
Biosafety evaluation of DOX@8py NPs. (A) Serum ALT, AST, BUN, CK, and CRE levels of LNCaP-tumor bearing mice after treatment with normal saline (control group), DOX (10 mg/kg), DOX@8p4 NPs, and DOX@8p6 NPs (equivalent DOX dose: 10 mg/kg). (B) Representative H&E images of the major organs extracted from LNCaP-tumor bearing mice at the end of the study. Scale bar: 200 μm.

Ensuring good biosafety is critical for the successful application of nanoformulations. Therefore, we performed a hemolysis test to assess the blood compatibility of the nanocarriers. As shown in Fig. S13, both 8p4 NPs and 8p6 NPs demonstrated favorable blood compatibility. Furthermore, following the treatment course, we evaluated the impact of different treatments via biochemical detection of blood and histopathological analysis of major organs. Quantitative analysis of biochemical indices, including alanine aminotransferase (ALT), aspartate transaminase (AST), creatinine (CRE), blood urea nitrogen (BUN), and creatine kinase (CK), is shown in Fig. 7A. We found that treatment with free DOX slightly elevated the levels of BUN, CK, ALT and AST, whereas DOX@8py NPs showed no upregulation in these indices, compared with the control group. Namely, DOX might cause nephrotoxicity and hepatotoxicity to some extent, but DOX@8py NPs could effectively mitigate such toxicity. Furthermore, H&E images from the major organs indicated that some pathological changes, such as minor congestion of myocardial fibers, nucleus dissolution, and disappearance were observed in the DOX group while no obvious structural damages or metabolic lesions were found in the control and DOX@8py NP groups (Fig. 7B). Therefore, these results suggested that Phe-PEA is a biosafe nanocarrier.

## Conclusion

In summary, we successfully developed a series of Phe-PEA polymers and utilized them to deliver DOX for prostate cancer therapy. The polymer structures of Phe-PEAs were finely tuned by changing the number of methylene groups in polymer backbone, which further led to differences in molecule weight, hydrophobicity, and thermal properties. We systemically evaluated the physicochemical properties and biofunctions of the corresponding DOX@PEA NPs. All the DOX@PEA NPs showed acceptable drug loading capacity, good stability, enhanced in vitro cytotoxicity, and efficient cellular uptake. More importantly, the systemic administration of DOX@PEA NPs significantly inhibited tumor progression and mitigated the side effects in mice, demonstrating the enhanced antitumor efficiency and biosafety of Phe-PEA for delivering DOX in vivo*.* Overall, these findings demonstrate the potential of Phe-PEA NPs as a promising drug delivery platform for prostate cancer therapy. More importantly, the finely tunable nature of Phe-PEA polymers and the successful development of Phe-PEA NPs as effective carriers for DOX delivery might inspire the development of Phe-PEA NPs for broader applications, facilitating their advancements in delivering various therapeutic agents and targeting different types of cancer.

## Materials and Methods

The materials and methods can be found in the Supplementary Materials.

## Data Availability

The data are available from the authors upon a reasonable request.

## Supplementary Materials

Supplementary 1 Figs. S1 to S13 Table S1 Materials and Methods section
