# Supplementary material for: Impact of Poly(Ester Amide) Structure on Properties and Drug Delivery for Prostate Cancer Therapy
Source: BME Front. 2023 Aug 10;4:0025. doi: 10.34133/bmef.0025 (PMC10414751; doi:10.34133/bmef.0025)
Supplement: Supplementary 1 — Figs. S1 to S13 Table S1 Materials and Methods section [file bmef.0025.f1.docx]

**Supporting Information**

**Impact of Poly(ester amide) Structure on Properties and Drug Delivery for Prostate Cancer Therapy**

Junfu Zhang ^a, †^, Liying Wang ^b, †^, Mengting Ding ^a^, Xinru You ^c^, Jun Wu ^d,e,*^, Jun Pang ^a,*^

*^a^ Department of Urology, Kidney and Urology Center, Pelvic Floor disorders Center, The Seventh Affiliated Hospital, Sun Yat-sen University, Shenzhen, 518107, China*

*^b^ Department of Hematology, The Seventh Affiliated Hospital, Sun Yat-sen University, Shenzhen, 518107, China*

*^c^ Center for Nanomedicine and Department of Anesthesiology, Brigham and Women's Hospital, Harvard Medical School, Boston, MA 02115, USA*

*^d^ Bioscience and Biomedical Engineering Thrust, The Hong Kong University of Science and Technology (Guangzhou), Nansha, Guangzhou, 511400, China*

*^e^ Division of Life Science, The Hong Kong University of Science and Technology, Hong Kong SAR, China*

**Experimental section**

**Materials and reagents**

Doxorubicin (DOX) was purchased from MedChemExpress. Different diol regents (1,2-ethanediol, 1,3-propanediol, 1,4-butanediol, 1,6-hexanediol, 1,8-octanediol), L-phenylalanine, toluene-4-sulfonic acid monohydrate, sebacoyl dichloride, p-nitrophenol and dimethylsulfoxide (DMSO) were obtained from Sigma-Aldrich. DSPE-PEG 2k was provided by Avanti Polar Lipids. MTT was purchase from Aladin. Annexin V-APC/7-AAD Apoptosis Detection Kit were purchased from MultiSciences. Hoechst 33342 and FITC-phalloidin were obtained from Invitrogen. Dulbecco's modified eagle medium (DMEM), penicillin/streptomycin (P/S), trypsin-EDTA, fetal bovine serum (FBS), and PBS solution were all purchased from Gibco.

**Cells and animals**

Prostate cancer LNCaP cells were obtained from Tianjin SaierBio and cultured following the recommended protocols. Cells were cultured using DMEM supplemented with 10% FBS and 1% penicillin P/S and incubated in an atmosphere with 5% CO_2_ under 37°C. The BALB/c nude mice (male, 4-5 weeks old) were obtained from Laboratory Animal Center of Sun Yat-Sen University. All the mice were bred in specific-pathogen-free facility with free access to water and food. The experiment protocols were approved by the Experimental Laboratory Animal Committee of Sun Yat-sen University (SYSU-IACUC-2021-000633).

**Synthesis and characterization of Phe-PEA polymers**

Phe-PEA polymers are composed of three major building blocks: phenylalanine, dicarboxylic acids and fatty diols. Briefly, the synthesis process of Phe-PEAs consisted of two primary stages: (1) the synthesis of two types of monomers—di-p-nitrophenyl ester of dicarboxylic acid (named “Nx”, “x” indicates the number of -CH_2_ in dicarboxylic acid; monomer I) and tetra-*p*-toluenesulfon acid salts of bis(L-Phe)-diester (named Phe-y, “y” indicates the number of -CH_2_ in diol; monomer Ⅱ); and (2) the synthesis of Phe-PEA polymers (named “xpy”) via rapid polycondensation of monomers I and II. The synthesis protocols of monomers and Phe-PEA were reported in previous studies[^30^](#_ENREF_30)^,^ [^32^](#_ENREF_32). In this study, di-*p*-nitrophenyl sebacate (N8) was chosen as monomer I and six types of monomer II were synthesized with “y” ranging from 2 to 8. The chemical structures of monomers were first confirmed by ^1^H -nuclear magnetic resonance (^1^H-NMR, Bruker, Switzerland). Via the combinations of monomers I and II, six types of Phe-PEA were finally synthesized (**Table S1**).

Next, the synthesized Phe-PEA polymers were characterized using different standard methods. Specifically, the chemical structures of these Phe-PEA polymers were determined by Fourier-transform infrared spectroscopy (FT-IR, Bruker, Germany) and ^1^H-NMR. For molecular weight (MW) characterization, Phe-PEA polymers was dissolved in tetrahydrofuran (1 mg/ml) and detected by gel permeation chromatography (GPC, Agilent, USA). Polystyrene with MW ranging from 20438 to 39659 was used as standards to calculate the MW. Additionally, to qualitatively evaluate hydrophobicity of polymers, fluorescent probe Nile red was encapsulated into polymers via precipitation method and measured by fluorescence spectrophotometer (Edinburgh, UK).

**Preparation and characterization of NPs**

The blank Phe-PEA carriers (8py NPs) and DOX-encapsulated Phe-PEA NPs (DOX@8py NPs) were both fabricated by a simple nanoprecipitation method. First, 8py polymer, DOX and DSPE-PEG 2k were separately dissolved in DMSO (20 mg/mL) and mixed at a mass ratio of 9:1:2. The mixture solution was slowly added into the stirring water. The formed NP solution was concentrated via ultrafiltration and suspended in PBS for further characterization and use. 8py NPs were fabricated in the absence of DOX and used as blank controls. Next, the properties of 8py NPs and DOX@8py NPs were characterized using various standard methods. Dynamic light scattering (DLS; Zetasizer Nano-ZS90, Malvern, UK) and transmission electron microscopy (TEM; JEM-200, JBOL, Japan) were applied to characterize the particle size and the morphology of blank NPs and DOX@8py NPs, respectively. Additionally, to assess the stability, 8py NPs and DOX@8py NPs were maintained in PBS (pH=7.4) and PBS containing 10% FBS, which were continuously measured by DLS for a week. The drug loading efficiency (DLE) and drug loading capacity (DLC) of DOX@8py NPs were detected by fluorescence spectrophotometry under excitation/emission (Ex/Em) wavelengths of 480/590 nm and calculated using the following equations:

$$DLE\%=\frac{mass of loaded drug}{mass of feeding drug}$$

$$DLC\%=\frac{mass of loaded drug}{mass of drug loaded NPs}$$

***In vitro* drug release**

DOX@8p4 NPs and DOX@8p6 NPs with relatively higher LC were screened to investigate the DOX release curves of DOX@8py NPs through dialysis method. Briefly, DOX@8py NPs were added into dialysis bags (MWCO: 3500) and immersed in PBS with different pH values (pH=5.4 and 7.4). The DOX release process was conducted in a shaker with a fixed shaking speed of 100 rpm under 37°C. At prearranged timepoints, the sample solution was withdrawn and the same amount of fresh release medium was supplemented. Finally, the amounts of DOX released from the NPs were analyzed by fluorescence spectrophotometry as mentioned above.

**Cytotoxicity assay**

The cytotoxicity of 8py NPs and DOX@8py NPs against LNCaP cells was assessed by MTT assay. LNCaP cells at a density of 5×10^3^ cells per well were plated in 96-well plates and cultured overnight. Next, the culture medium was removed and the cells were treated with new medium containing blank 8py NPs, DOX, or DOX@8py NPs across different concentrations for 24 h. Then, MTT solution was added to treat LNCaP cells for another 4 h. Next, the medium was removed and DMSO was added to thoroughly dissolve the formed formazan. Finally, the absorbance of each well at 490 nm was detected using microplate reader (Synergy4, Bio Tek, USA). The cell viability was determined as follows:

$$Cell viability\%=\frac{\mathrm{Ab}_{\mathrm{sample}}-\mathrm{Ab}_{\mathrm{blank}}}{\mathrm{Ab}_{\mathrm{control}}-\mathrm{Ab}_{\mathrm{blank}}}\times100\%$$

**Apoptosis assay**

For cell apoptosis, LNCaP cells at a density of 2×10^5^ cells per well were plated in 6-well plates and cultured overnight. Next, the culture medium was discarded, and the new medium supplemented with DOX and DOX@8py NPs at an equivalent concentration of 1 μg/mL was added to treat cells for 24 h. Then, LNCaP cells were digested by trypsin without EDTA and further collected by centrifugation, followed by staining with 7-AAD and Annexin V-APC away from the light for 15 min. Finally, the apoptotic cells induced by drugs were detected by flow cytometry (Sony, Japan).

**Cellular uptake and internalization**

Next, the cellular uptake of DOX@8py NPs on prostate cancer cells was investigated by flow cytometer. LNCaP cells at a density of 20×10^5^ cells per well were plated in 6-well plates and cultured overnight. The cells were the incubated with DOX and different DOX@8py NPs at an equivalent concentration of 1 μg/mL for 1, 4, 8 and 12 h. Following this, the cells were collected for flow cytometer analysis.

Furthermore, DOX@8p4 NPs and DOX@8p6 NPs were chosen to determine the cellular internalization of DOX@8py NPs on prostate cancer cells. LNCaP cells at a density of 1×10^5^ cells per well were plated in glass-bottom dishes and cultured overnight. The cells were then treated with DOX@8p4 NPs and DOX@8p6 NPs at an equivalent concentration of 2 μg/mL for 1, 4, 8, and 12 hours. Subsequently, the cells were washed twice with cold PBS and stained with Hoechst 333425 (a nuclear counterstain, 5 μg/mL) for 10 min and FITC-phalloidin (a cytoskeleton actin marker, 100 nM) for 30 min. Finally, LNCaP cells were imaged using confocal laser scanning microscopy (CLSM; Olympus, Japan).

Meanwhile, to quantitatively determine the real DOX uptake level, LNCaP cells at a density of 5×10^5^ cells per well were plated in 6-well plates and cultured overnight. Next, DOX@8p4 NPs and DOX@8p6 NPs at an equivalent concentration of 2 μg/mL were added to incubate with cells for 1, 4, 8 and 12 h. After that, RIPA lysis buffer was added to lyse the cells and the cell lysates were centrifuged at 12000 rpm for 15 min. Next, the obtained supernatant was mixed with trichloromethane and vigorously vortexed for 5 min to extracted DOX. After centrifugation at 12000 rpm for 5 min, the substratum was collected and blow-dried under a stream of nitrogen. Next, 200 μL of mobile phase (water with 0.01% phosphoric acid/acetonitrile= 70/30) was added to redissolve the drug. After centrifugation at 12000 rpm for 15 min, 20 μL of supernatant was injected for HPLC-FLD (Shimadzu, Japan) analysis with excitation/emission wavelength set to 480/590 nm. Finally, the cellular DOX levels were normalized to protein concentration.

**Hemolysis test**

To evaluate the blood compatibility of NPs, 8p4 NPs and 8p6 NPs across different concentrations were mixed with red blood cell suspension and incubated in a constant temperature shaking table at 37°C and 100 rpm for 3 h. PBS mixed with the red blood cell suspension was set as the negative control group, while samples with ultra-pure water mixed with the red blood cell suspension were set as the positive control group. After incubation, the samples were centrifuged at 1000 rpm for 5 min and the supernatant was collected to determine the absorbance at 540 nm. The hemolysis rate was determined as follows:

Hemolysis%=(Ab_sample_-Ab_negative_)/(Ab_positive_-Ab_negative_)×100%

***In vivo* antitumor efficacy**

The *in vivo* antitumor efficacy of DOX@8py NPs was assessed in a human prostate cancer xenograft model. The model was constructed by subcutaneously injecting LNCaP cell suspension into the back region of nude mice. As the tumor size increased to ≈ 100 mm^3^, the tumor-bearing mice were randomized into 4 groups, and intravenously administered with PBS, DOX (10 mg/kg), DOX@8p4 NPs (DOX dose:10 mg/kg), and DOX@8p6 NPs (DOX dose:10 mg/kg) every 3 days. The body weight and tumor volume of the mice were monitored every 2 days. Specifically, the tumor volume was determined as follows:

$$Volume (\mathrm{mm}^{3})=\frac{length\times\mathrm{width}^{2}}{2}$$

**Biosafety evaluation**

The *in vivo* biosafety of DOX@8py formulations was assessed by biochemical and histopathological detection. Briefly, blood samples were collected from the orbits of mice in each group at the end of the treatment period and detected by a HITACHI Automatic Aralyzer (Japan). Next, the tumor tissues and major organs were resected from the sacrificed mice, followed with fixation in 4% paraformaldehyde and paraffin embedding. Slides of major organs and tumor tissues were stained with hematoxylin and eosin (H&E) and photographed using a fully automated upright microscope (Leica, Germany). Additionally, TUNEL assay was performed to investigate the apoptotic cells in tumor tissues.

**Statistical Analysis**

The experiment results were expressed as mean ± SD and repeated in triplicate. Two-tailed Student’s t-test was performed to determine the statistical significance the difference between two groups, and one-way analysis of variance (ANOVA) was performed to determine the statistical significance of multiple groups. **P*< 0.05, ***P*< 0.01, ****P*<0.001.

**Table S1.** Combinations of Phe-PEA polymers and their property characterization results.

| Monomer I | Monomer II | Polymer | Mn | Mw | PDI (Mw/Mn) | Tg (℃) |
| --- | --- | --- | --- | --- | --- | --- |
| N8 | Phe-2 | 8p2 | 29917 | 39659 | 1.8474 | 20.4 |
| N8 | Phe-3 | 8p3 | 21161 | 42409 | 2.0041 | 42.2 |
| N8 | Phe-4 | 8p4 | 30870 | 36159 | 1.3674 | 19.1 |
| N8 | Phe-5 | 8p5 | 24822 | 50940 | 2.0522 | 33.6 |
| N8 | Phe-6 | 8p6 | 10726 | 20438 | 1.9055 | 19.3 |
| N8 | Phe-8 | 8p8 | 15052 | 27513 | 1.8279 | 21.9 |

Mn: number-average molecular weight; Mw: weight-average molecular weight; PDI: polydispersity index; Tg: glass transition temperature.


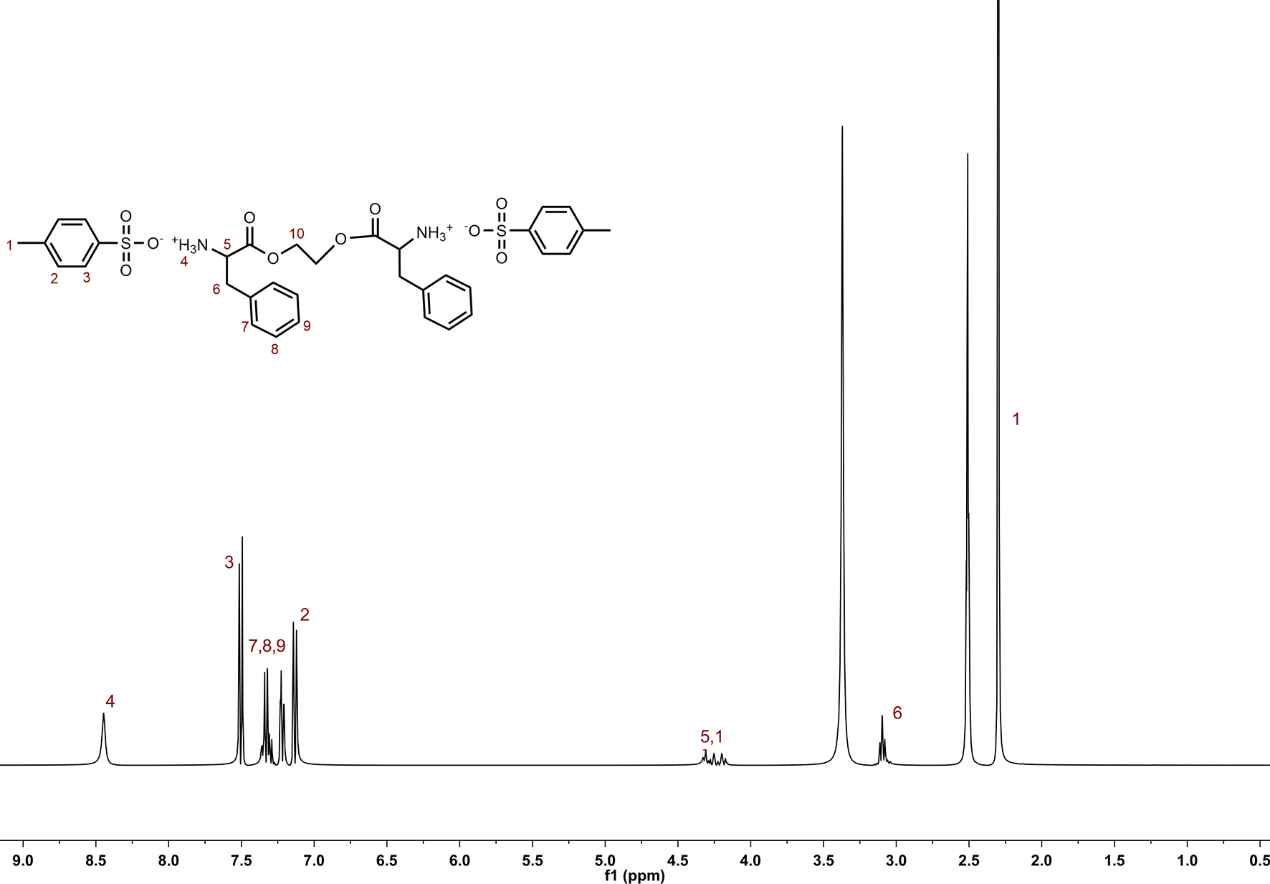


**Fig. S1** ^1^H-NMR spectrum of Phe-2.


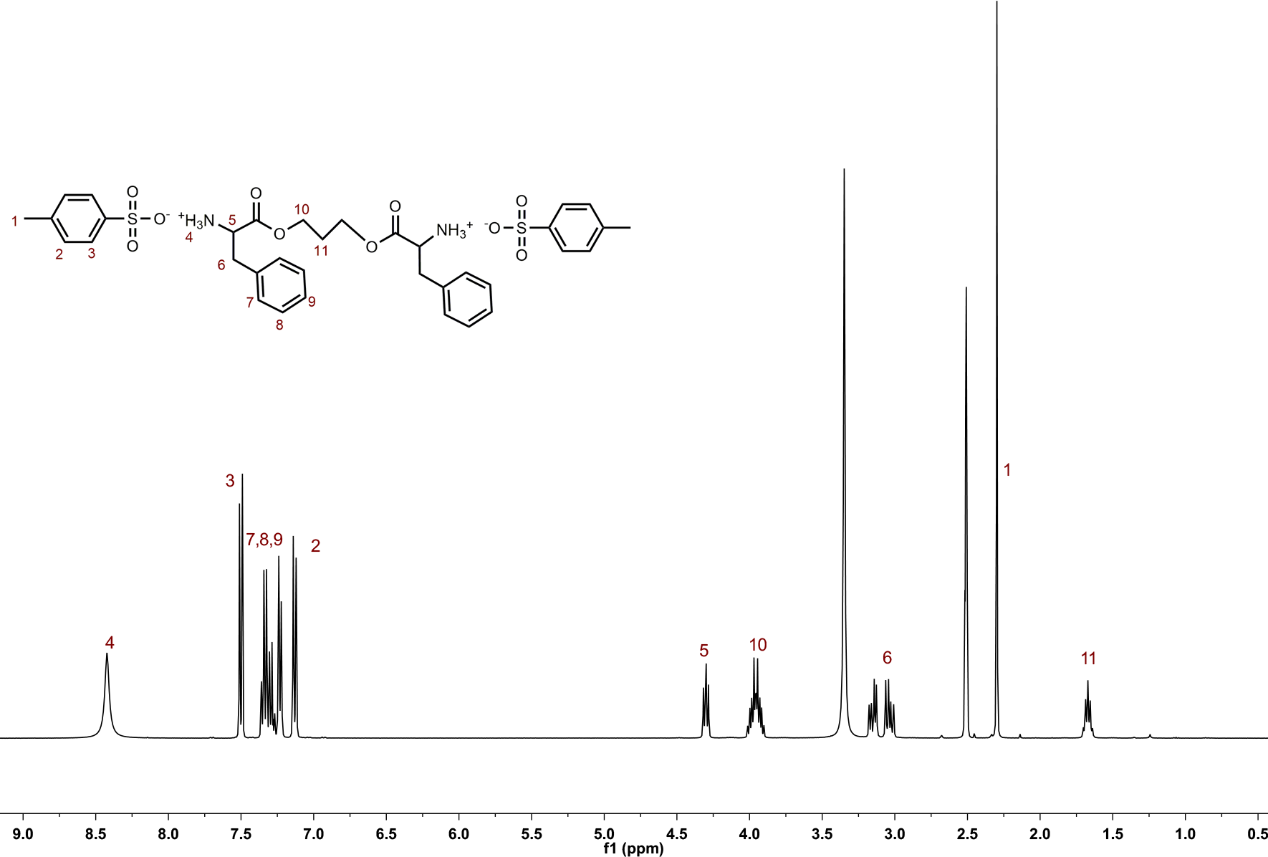


**Fig. S2** ^1^H-NMR spectrum of Phe-3.


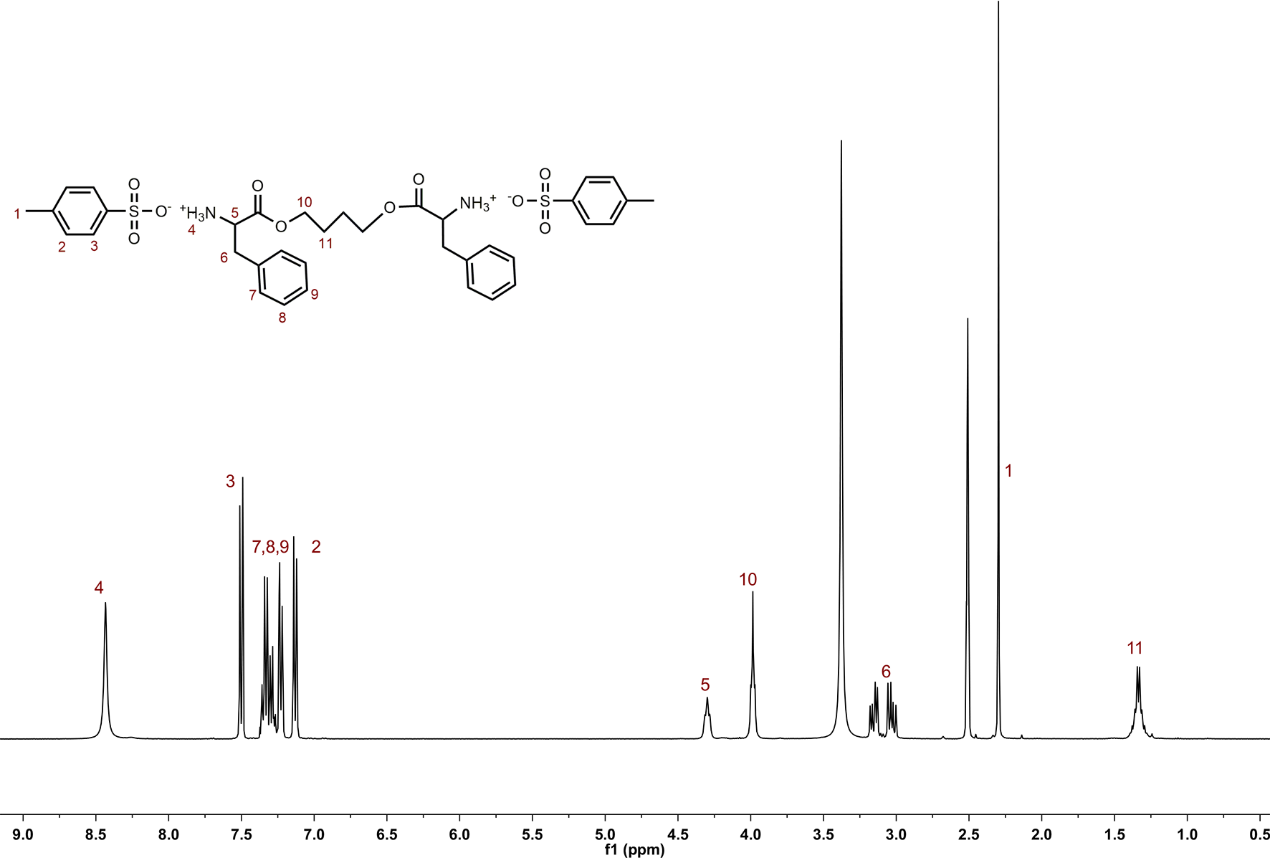


**Fig. S3** ^1^H-NMR spectrum of Phe-4.


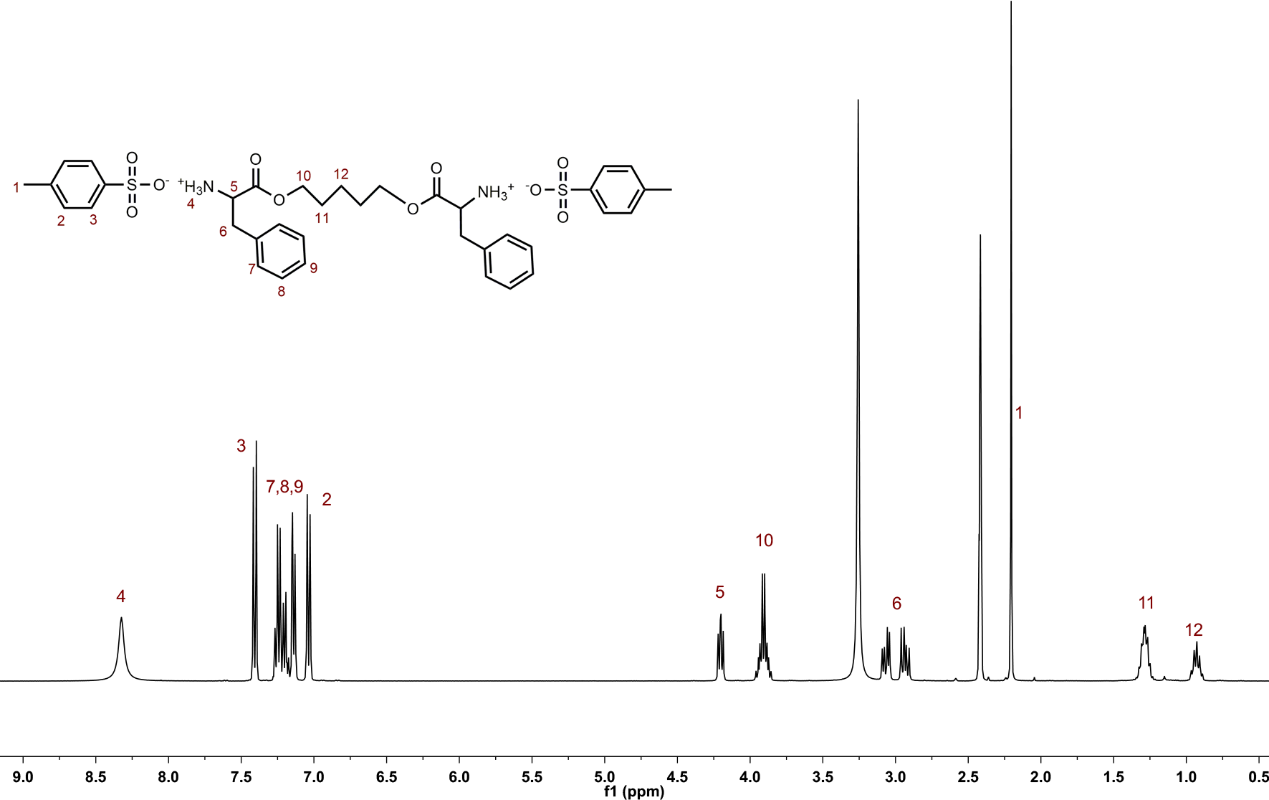


**Fig. S4** ^1^H-NMR spectrum of Phe-5.


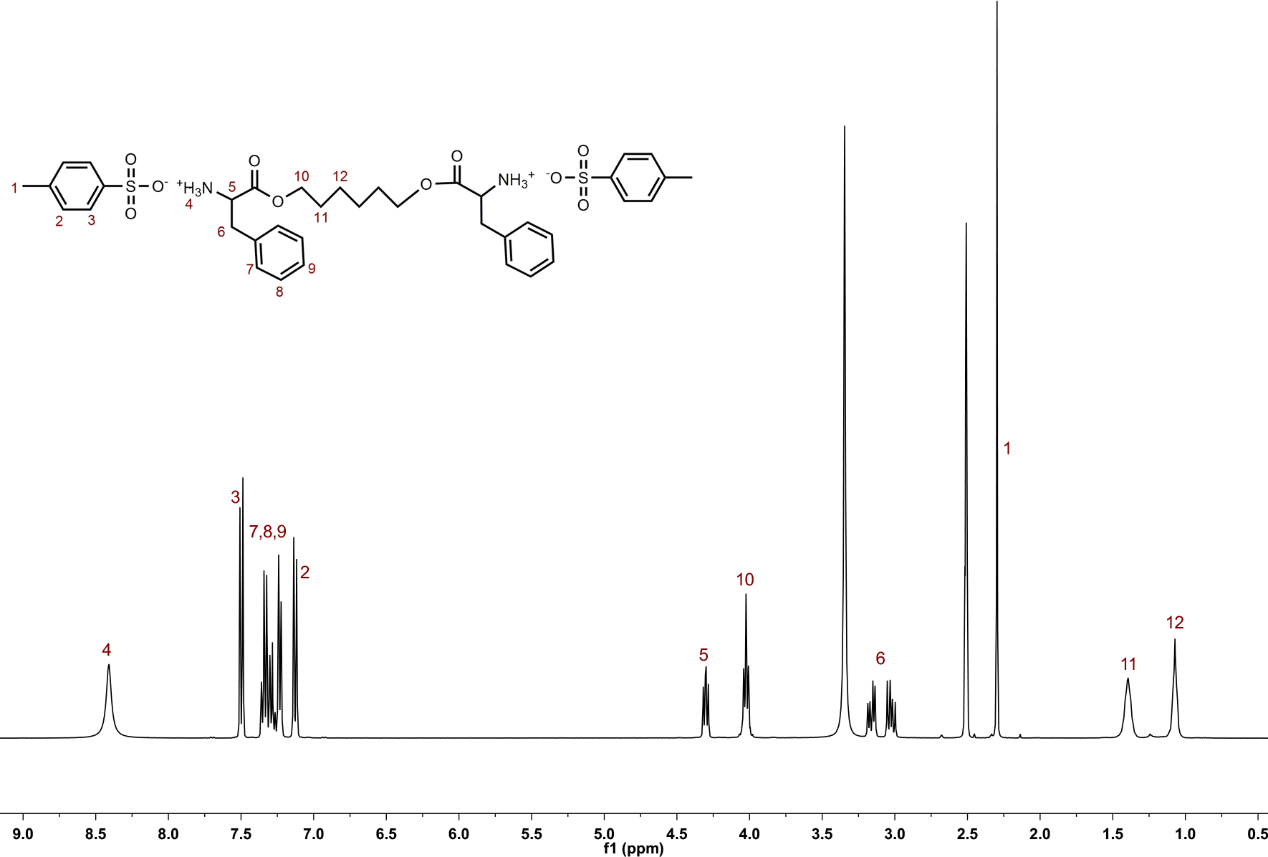


**Fig. S5** ^1^H-NMR spectrum of Phe-6.


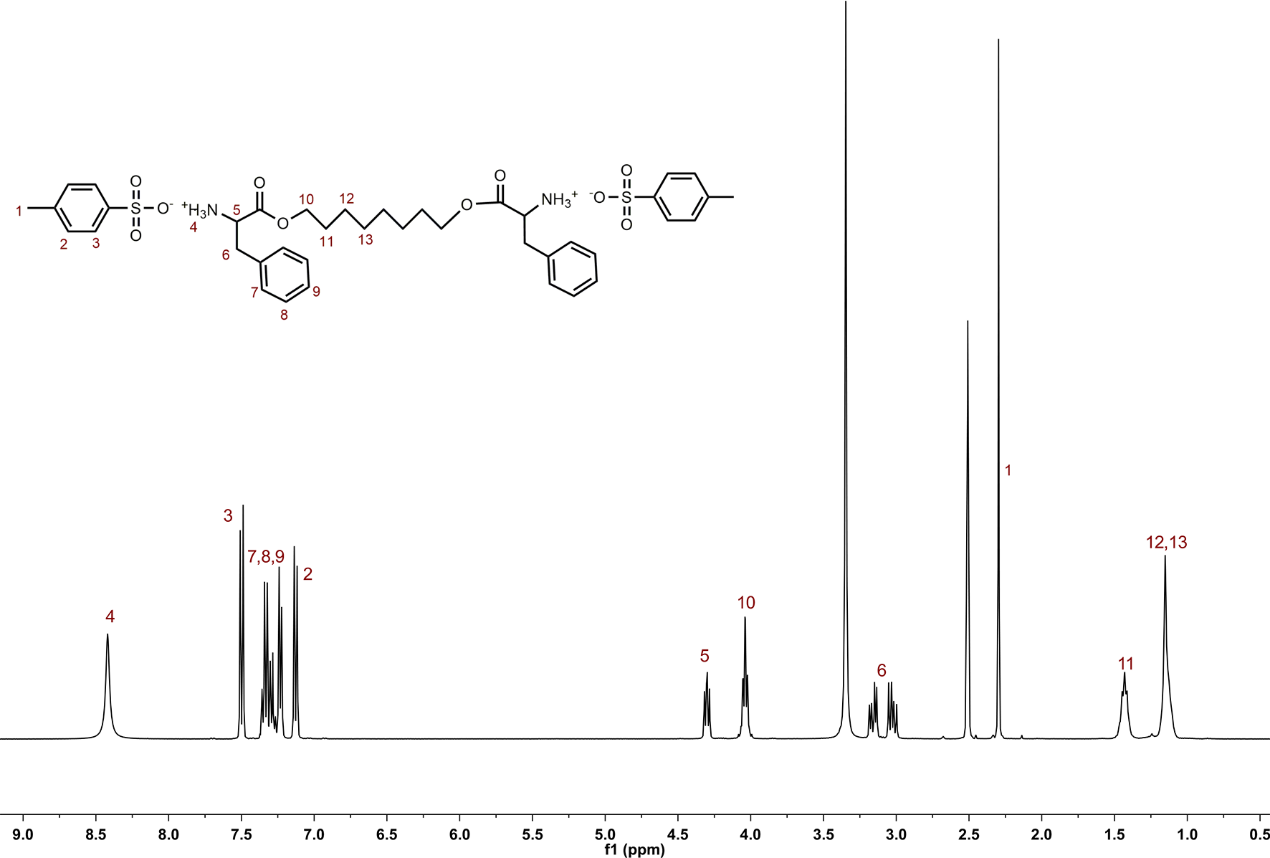


**Fig. S6** ^1^H-NMR spectrum of Phe-8.


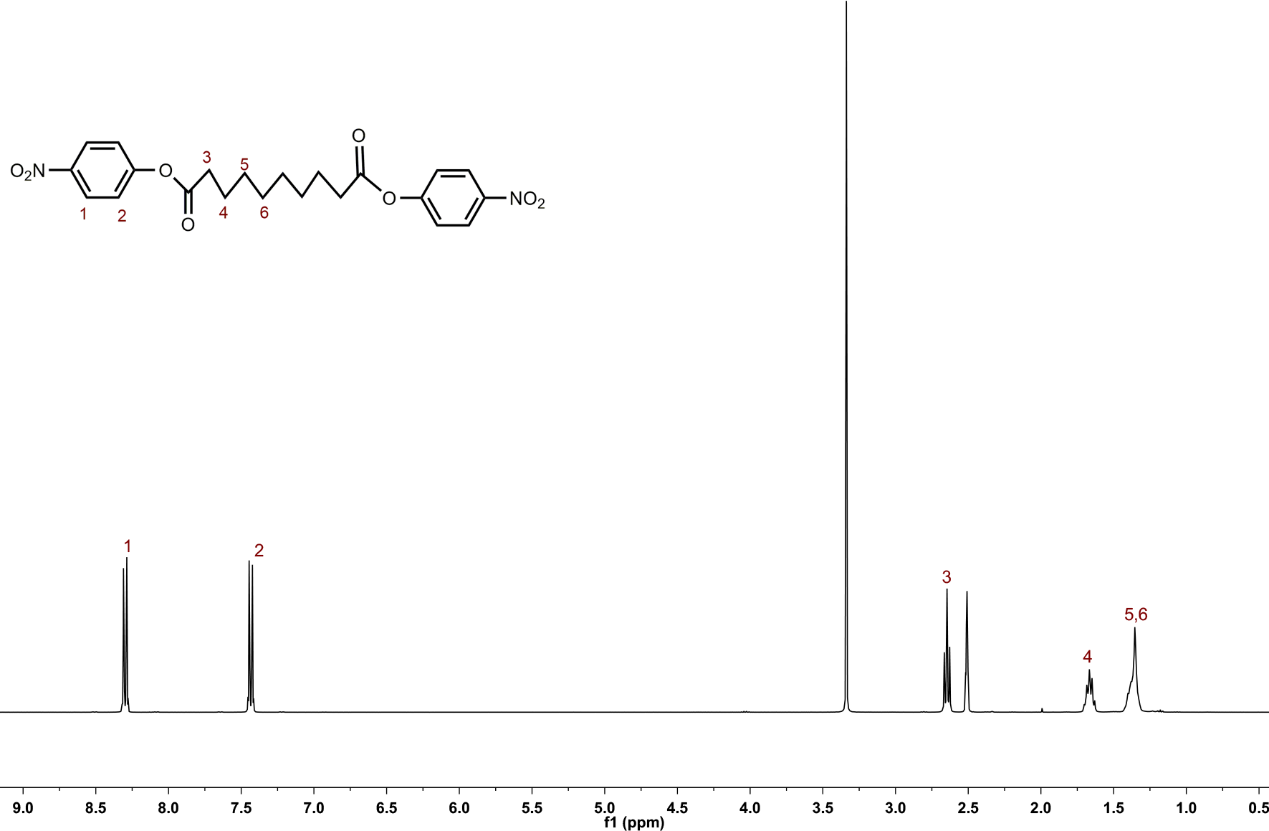


**Fig. S7** ^1^H-NMR spectrum of N8.


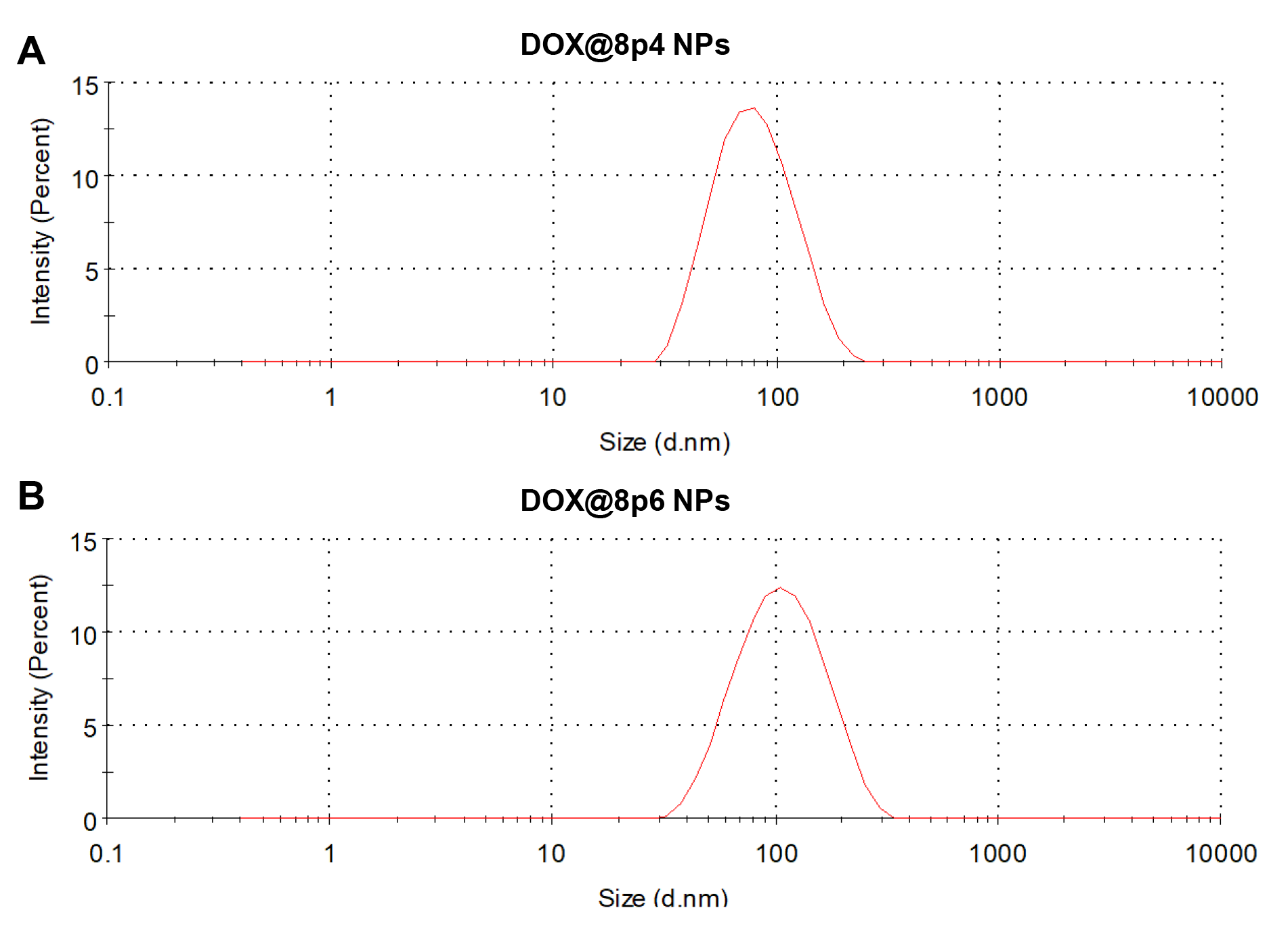


**Fig. S8** Size distribution of (A) DOX@8p4 NPs and (B) DOX@8p6 NPs.


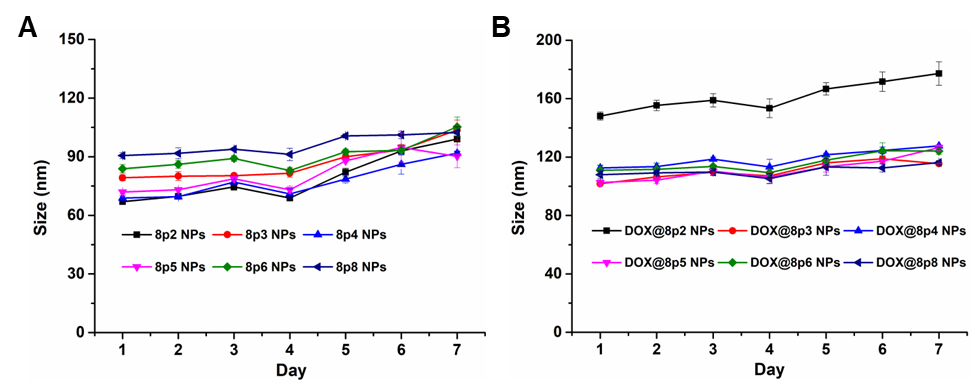


**Fig. S9** Stability test of (A) 8py NPs and (B) DOX@8py NPs in PBS with 10% FBS for 7 days.

**
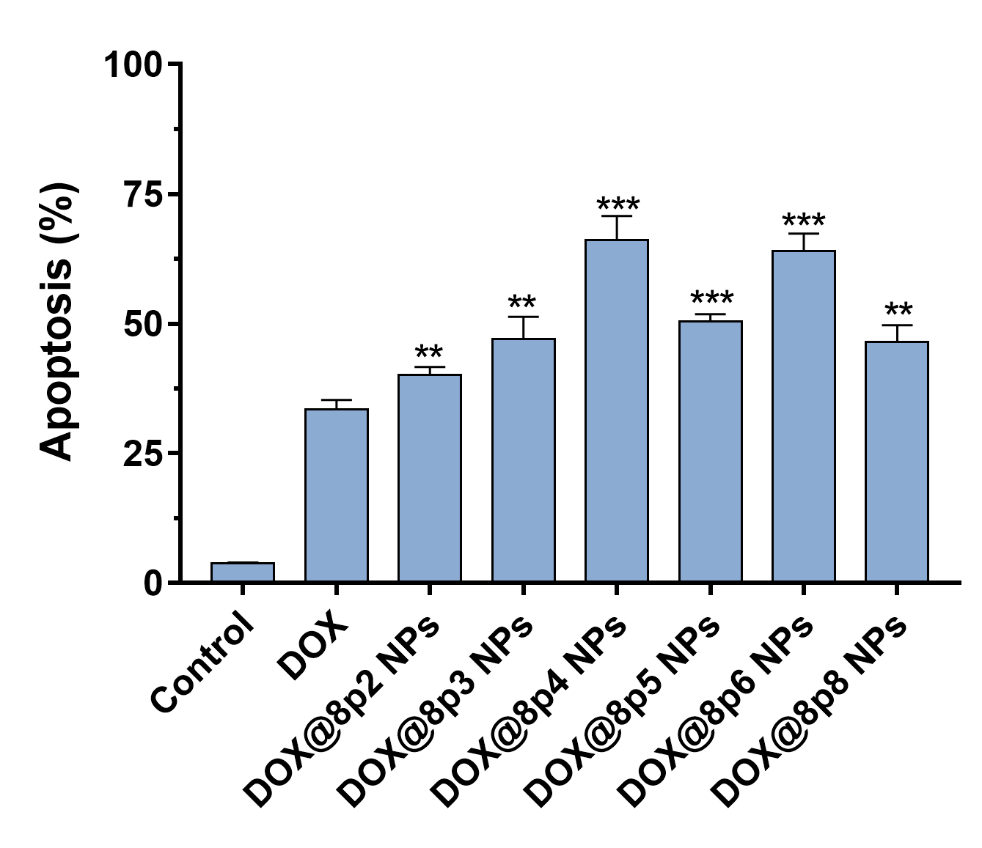
**

**Fig. S10** Quantitative analysis for the apoptosis of LNCaP cells induced by DOX and DOX@8py NPs. (***P*< 0.01 vs DOX, ****P*< 0.001 vs DOX)


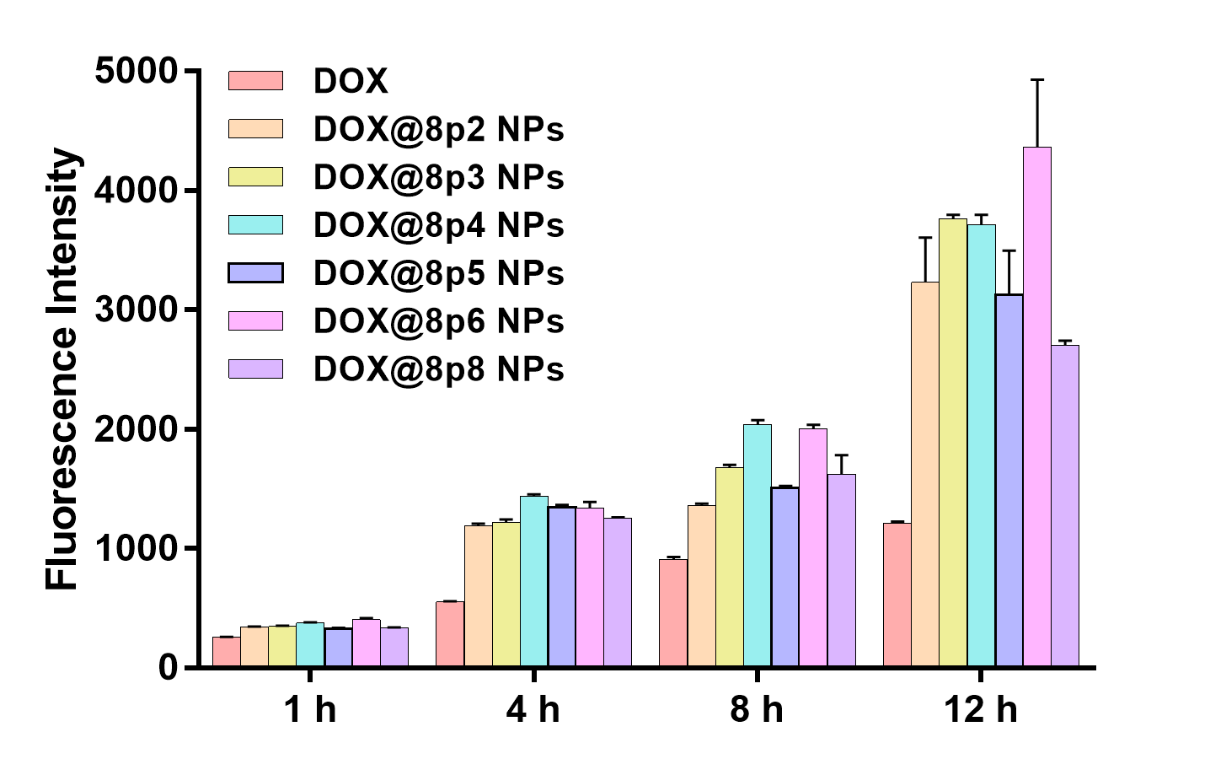


**Fig. S11** Quantitative analysis of cellular uptake detected by flow cytometry.


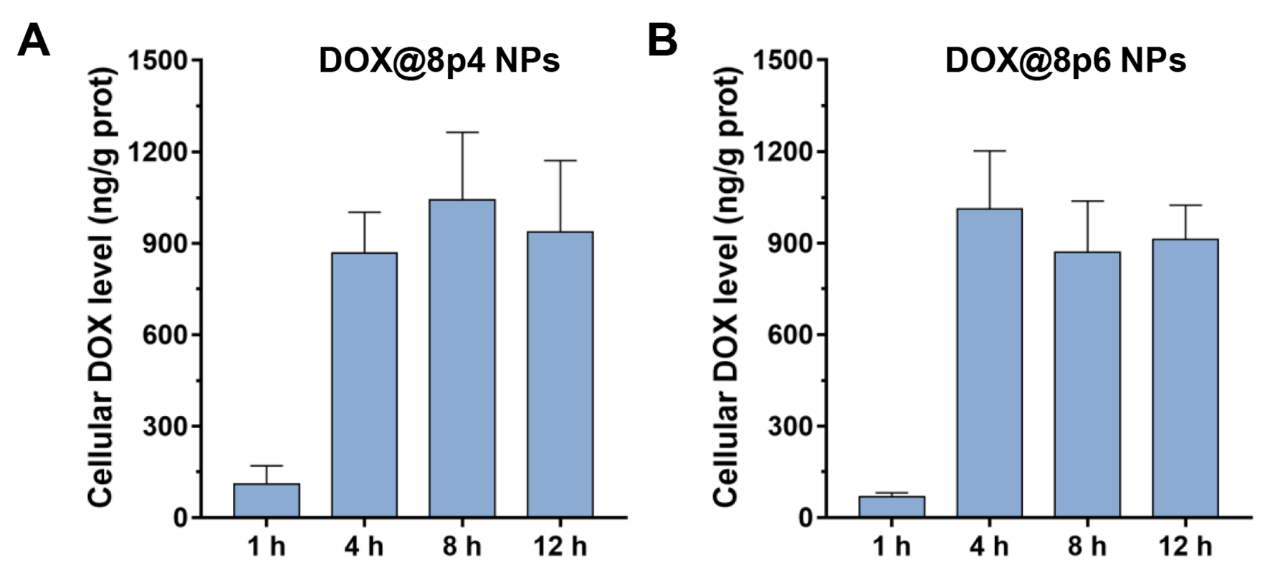


**Fig. S12** Cellular DOX level analyzed by HPLC-FLD after incubation with DOX@8p4 NPs (A) and DOX@8p6 NPs (B). g prot, g protein.


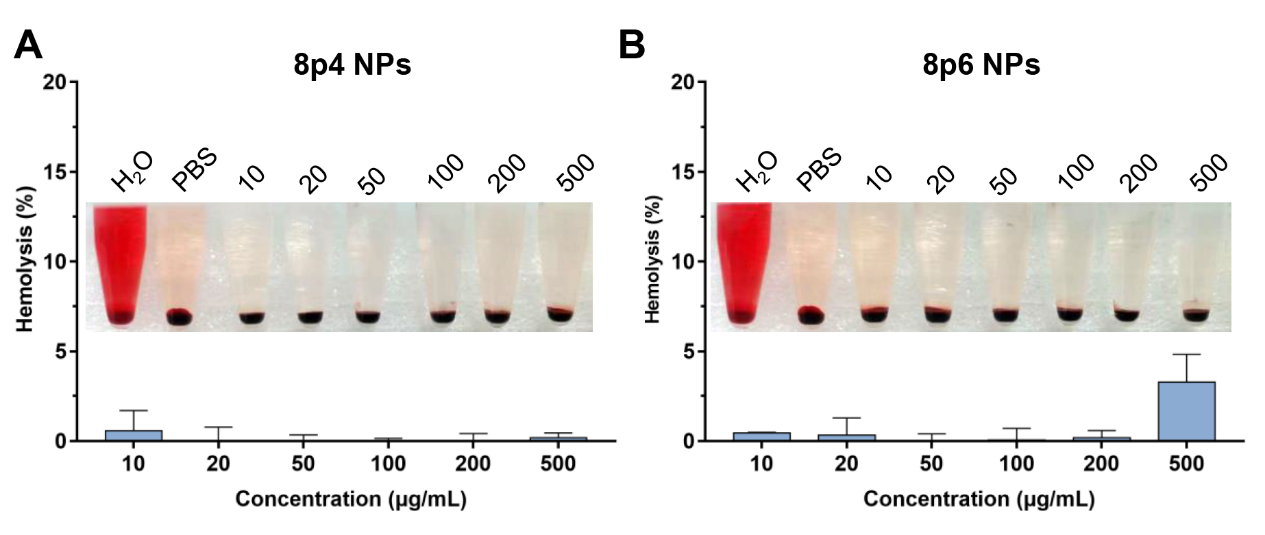


**Fig. S13** Hemolysis rate of (A) 8p4 NPs and (B) 8p6 NPs across different concentrations.
